# Sensing the (digital) pulse. Future steps for improving the secondary use of data for research in Switzerland

**DOI:** 10.1177/20552076231169826

**Published:** 2023-04-20

**Authors:** Andrea Martani, Lester Darryl Geneviève, Tenzin Wangmo, Julia Maurer, Katrin Crameri, Frédéric Erard, Julia Spoendlin, Christiane Pauli-Magnus, Valerie Pittet, Thierry Sengstag, Emiliano Soldini, Bernard Hirschel, Bettina Borisch, Cornelia Kruschel Weber, Marcel Zwahlen, Bernice Simone Elger

**Affiliations:** 1 Institute for Biomedical Ethics, 27209 University of Basel, Basel, Switzerland; 2 Personalized Health Informatics Group, 30489 SIB Swiss Institute of Bioinformatics, Basel, Switzerland; 3 Legal & Technology Transfer, 30489 Swiss Institute of Bioinformatics (SIB), Lausanne, Switzerland; 4 Basel Pharmacoepidemiology Unit, Division of Clinical Pharmacy and Epidemiology, Department of Pharmaceutical Sciences, 27209 University of Basel, Basel, Switzerland; 5 Hospital Pharmacy, 30262 University Hospital Basel, Basel, Switzerland; 6 Clinical Trial Unit, Department of Clinical Research, University of Basel and University Hospital Basel, Basel, Switzerland; 7 Center for Primary Care and Public Health, Department of Epidemiology and Health Systems, 27213 University of Lausanne, Lausanne, Switzerland; 8 sciCORE, 27209 University of Basel, Basel, Switzerland; 9 Competence Centre for Healthcare Practices and Policies, Department of Business Economics, Health and Social Care, University of Applied Sciences and Arts of Southern Switzerland, Manno, Switzerland; 10 Cantonal Ethics Commission for Research on Human Beings, Geneva, Switzerland; 11 Institute of Global Health, 27212 University of Geneva, Geneva, Switzerland; 12 Independent Researcher, Zürich, Switzerland; 13 Institute of Social and Preventive Medicine, 27210 University of Bern, Bern, Switzerland; 14 University Center of Legal Medicine, 27212 University of Geneva, Geneva, Switzerland

**Keywords:** Health data policy, Switzerland, secondary use of data, health informatics, ELSI, digital health

## Abstract

**Introduction:**

Ensuring that the health data infrastructure and governance permits an efficient secondary use of data for research is a policy priority for many countries. Switzerland is no exception and many initiatives have been launched to improve its health data landscape. The country now stands at an important crossroad, debating the right way forward. We aimed to explore which specific elements of data governance can facilitate – from ethico-legal and socio-cultural perspectives – the sharing and reuse of data for research purposes in Switzerland.

**Methods:**

A modified Delphi methodology was used to collect and structure input from a panel of experts via successive rounds of mediated interaction on the topic of health data governance in Switzerland.

**Results:**

First, we suggested techniques to facilitate data sharing practices, especially when data are shared between researchers or from healthcare institutions to researchers. Second, we identified ways to improve the interaction between data protection law and the reuse of data for research, and the ways of implementing informed consent in this context. Third, we put forth ideas on policy changes, such as the steps necessary to improve coordination between different actors of the data landscape and to win the defensive and risk-adverse attitudes widespread when it comes to health data.

**Conclusions:**

After having engaged with these topics, we highlighted the importance of focusing on non-technical aspects to improve the data-readiness of a country (e.g., attitudes of stakeholders involved) and of having a pro-active debate between the different institutional actors, ethico-legal experts and society at large.

## Background

One central topic in the health policymaking agenda in Europe and beyond is to catalyse the progress permitted by digitalisation.^1^ At a supranational level, the European Union has made the creation of a European Health Data Space a priority to “promote better exchange and access to different types of health data (electronic health records, genomics data, data from patient registries etc.), not only to support healthcare delivery (so-called primary use of data) but also for health research and health policy making purposes”.^2^ At the national level, countries all over the world have already been investing in an effort to bring their healthcare systems into the digital era for a long time, but with different levels of commitment and results. Indeed, a cross-country comparative survey on this topic published in 2021 by the OECD clearly showed that some countries have already achieved substantial results in terms of health data infrastructure and governance (e.g., Denmark, Finland, and South Korea), but many others lag considerably behind.^3^

In Switzerland, the work on improving the health data infrastructure and governance started relatively late (the first federal e-health policy was launched in 2007, as compared to 1995 in Finland^4^) and it now stands at an important crossroad. On the one hand, some onerous infrastructural projects have recently become operative. For example, in the field of patient care, electronic platforms offering citizens the possibility to open up an interoperable Electronic Patient Dossier (EPD) are available since 2021 in many more regions,^5^ and they aim – in the near future – to allow patients to save their healthcare data and make them accessible to participating healthcare providers across the country.^6^ For what concerns secondary use of data for research purposes, the Swiss Personalised Health Network (SPHN) – a national initiative established to facilitate the analysis and exchange of health data collected mainly from patients treated in Swiss university hospitals – has obtained some success, e.g., by establishing data management platforms at the five university hospitals, implementing a national interoperability strategy and creating a Data Coordination Centre to facilitate the usability of clinical data for research purposes^1^. Moreover, there are several cohort studies, which have developed their own databases and set out procedures on how researchers can access them.^7,8^ On the other hand, there are many doubts on the future steps for several elements of the Swiss health data infrastructure, both concerning how to guarantee financial sustainability and how to ensure that they develop in a scalable and useful manner for stakeholders. In the field of data reuse in clinical care, it has recently been proposed^9^ that all healthcare providers will have to be connected to the EPD infrastructure, and that data therein contained should be made available for reuse in the research context, but the physicians’ professional association have showed opposition to these development.^6,10^ Also in respect to the elements of the data infrastructure aimed primarily at facilitating data reuse for biomedical research there have been issues. For example, the scalability and sustainability future of the infrastructure components developed as part of SPHN are uncertain, since the major funding streams for the initiative are expected to stop at the end of 2024, although the maintenance of established infrastructures is under discussion.^11^

Being at this crossroad, Switzerland is in the process of implementing a plan for the future development of its health data landscape for many purposes, ranging from improving clinical care to facilitating biomedical research. This remains a national priority in health policy, as highlighted in “Health2030”, a strategic document adopted by the Federal Council containing the vision for the future of healthcare in the country. Furthermore, the challenges posed by the COVID-19 pandemic have highlighted additional areas where improvement in data management is needed. In January 2022, the Federal Office of Public Health published a report concerning the future of data management in the field of health based on the lessons learned since the beginning of the COVID-19 pandemic.^12^ The report acknowledged some progress made during the pandemic (e.g., concerning the transmission of data from healthcare institutions to public health administrative units), but it also identified persisting shortcomings. It highlighted, for example, that a comprehensive strategy for the governance of data-flows in the health sector is missing and that the (re)use of data for research purposes remains in need of improvement, especially concerning the clarity of the data protection legal framework. Indeed, the issue of legal uncertainty hindering cross-cantonal or nationwide data sharing activities was also raised in one previous study we conducted. Contributors to this situation include not only the fact that Swiss researchers are sometimes ill-equipped to navigate through the multitude of cantonal data protection regulations and the federal data protection law, and consequently preferred to err on the side of caution by restricting data sharing activities, but also the multitude of regulatory authorities (e.g., cantonal data protection officers, research ethics committees, and federal institutions) having different interpretations of the same piece of legislation.^13^

With this article, we aim to contribute to the discussion on the next steps to take in the Swiss health data landscape at this important crossroad of its evolution. We do so by reporting the results of a study which we conducted with a modified Delphi methodology. Our aim was to explore which specific elements of data governance can facilitate – from ethico-legal and socio-cultural perspectives – the sharing and reuse of data for research purposes in Switzerland. This is a relevant policy issue in the field of digital health also for other countries, as demonstrated – for example – by the fact that one of the main objectives behind the proposal for creating a health data space across Europe is indeed that of easing the secondary use of data.^14^

## Methods

### Study design and methodology

This study is embedded in a larger project called SMAASH^15^ investigating the Swiss health data landscape and its future developments, with a particular focus on ethical and governance aspects. As part of this larger project, several activities were conducted, including: a systematic review to identify the main barriers and facilitators to health data processing and harmonisation;^16^ analyses of the interplay between data protection law and data sharing;^17,18^ analyses of how ethics bears on the exchange of health data;^19,20^ and interviews with more than 60 stakeholders practically involved in the health data infrastructure from both Switzerland and Denmark.^13,21,22^ As a result of these activities, we gathered many insights on the state of the Swiss health data landscape, on the key challenges at the important crossroad where it stands, and on potential ways how to tackle them. To further contribute to the policy debate in our context, as well as to inform the policymaking of other countries who face similar issues in the field of digital health, we aimed at specifying and narrowing down the scope of proposals how to concretely facilitate the secondary use of data for research. Therefore, to complement our previous work and answer remaining open questions, we set up the current study, which is based on a modified Delphi methodology. At its core, this methodology consists in creating a communication structure where experts from different specialties contribute information or judgements to a problem area which is broader in scope than the knowledge that every single individual in the group possesses.^23^ An essential feature of Delphi methodology is the iterative approach, meaning that information should be gathered from experts and then fed back to them, with the objective of inducing reflection in an environment of controlled interaction. The methodology also requires to maintain some degree of anonymity in the iterative interaction between experts, to limit reciprocal influencing amongst them. Finally, the Delphi approach is generally aimed at finding consensus on key elements to bring forward the topic analysed, or to elicit possible alternatives.

In the concrete implementation of the methodology, we adapted some aspects of the traditional Delphi methodology as described by Linstone and Turoff.^23^ Adapting the Delphi methodology is a common approach, as highlighted by McKenna who introduced the overarching label of “Modified ‘Delphi’” to cover the several adaptations of the technique available in the literature.^24^ Further labels have been suggested to describe the variations within the Modified Delphi, including ‘Reactive Delphi’,^24^ “Policy Delphi”^25^ or “Decision Delphi”.^26^ With Rauch, we share the idea that “[the different Delphi models] are only ‘ideal patterns’, which usually do not find counterpart in reality [and] every practical Delphi application is, in fact, a mixture”. This is true also for our application of the Delphi technique, which included some elements of the “Reactive Delphi” (e.g., asking participants to interact with previously prepared statements, rather than generating their own), and some of the Policy Delphi (e.g., not a clear commitment to reach consensus, but also to explore different positions and their pros and cons) and of the Decision Delphi (e.g., abandoning commitment to full anonymity). Therefore, whilst a classical Delphi typically involves several rounds of questionnaires where participants identify relevant issues and then anonymously rate their agreement and disagreement with them, our study included: (1) a preliminary questionnaire for experts on the topic of our study; (2) a two-day workshop with three round-tables for further discussion; (3) and a fed-back session on the result of the discussion after the workshop. Further details on each of these phases are described below.

### Participant selection and setting

To select participants for our study, we relied on purposive sampling by exploiting the network of experts our core team (AM, BSE, LDG and TW) had identified for the overarching project where this study is embedded. The aim was to involve a maximum of 15 experts, in order to facilitate in-person participation at a two-day workshop. Recruitment was complicated due to the COVID-19 pandemic, especially since our target group were all experts active in and around the healthcare sector. In total, 29 people were invited: 12 declined due to overlapping commitments, 3 did not reply, and 14 agreed to participate. One participant withdrew last minute for personal issues. The main^2^ expertise of the participants were: epidemiology (*n*=3), clinical research (*n*=2), data science and health informatics (*n*=2), global health (*n*=1), health services research (*n*=1), research ethics (*n*=1), ELSI (Ethical, Legal, and Social Issues) in health informatics (*n*=1), data protection Law (*n*=1), and policymaking in healthcare (*n*=1).

The workshop took place on 14^th^–15^th^ June 2021 in Hermance (Switzerland) at the Brocher Foundation, an institution nurturing research around current challenges in healthcare, with a specific focus on ethical, legal and social issues.

### Data collection

Before the workshop, we sent to the invitees a questionnaire tackling the major topics that we wanted to address in the workshop. The questionnaire was built based on the previous findings of SMAASH. Indeed, as part of this project, our core team (AM, BSE, LDG, TW) had already conducted several studies (see above in the section “Study design and methodology”), as well as interacted with numerous stakeholders of the Swiss health data landscape by being embedded in National Research Program 74 “Smarter Healthcare”. This program fostered and facilitated the interaction between researchers, policymakers and professionals of the health system,^27^ and one of the main pillars of this discussions centred around health care data.^28^ Based on our previous findings and on the participation in this activities, the structure and content of the questionnaire was determined by the core team through iterative meetings in which the structure, content and single items were debated. Some of the items were derived directly from the previous empirical research conducted as part of SMAASH, whereas others were derived from a reflexive analysis of the literature (the possibility of using Theory/Literature based statements in a Delphi is documented in the literature^29^). The workshop started by a presentation of the summary results of the SMAASH and the display of other material prepared by our core team. The latter included: (1) a video with a Danish epidemiologist, in which the structure of the advanced Danish health data landscape was illustrated, as a source of inspiration for developing solutions for the Swiss context; (2) an interview with a Swiss and a Danish epidemiologist was shown, in which they discussed more in details what each health data system could learn from the other, and what strategies could be adopted to improve the situation. Afterwards, we had three roundtable discussions on three specific topic-areas, in which panellists could provide their perspectives on priorities for advancing the Swiss health data landscape in the future. To stimulate discussion during these roundtables, we presented the aggregated responses from our preliminary questionnaire. Discussions were moderated by our core team and were aimed at trying to find consensus around the most important matters for the future of the Swiss health data landscape. Apart from discussing the items of the preliminary questionnaire, participants were invited to raise new items for discussions, and thus identify potential blind-spots. The discussions during the three roundtables were audio-recorded with the agreement of the participants, for a total of almost 5 hours of documentation. Moreover, we took field notes whilst the discussions were happening.

### Data analysis

**Figure 1.**
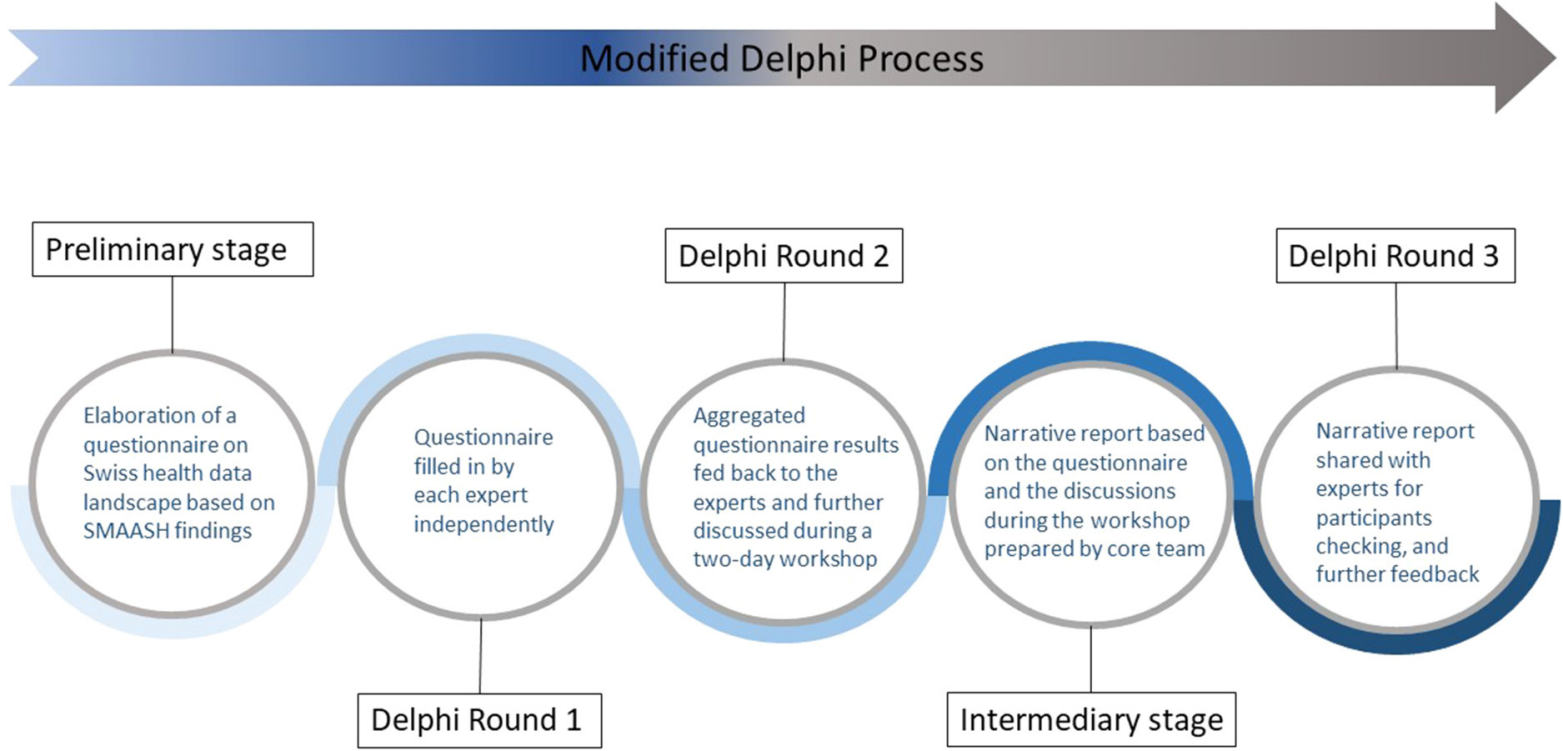
Structure of the modified Delphi process.

Combining the transcribed recordings and the field notes, we prepared a narrative summary of each of the three roundtables, i.e., we noted down the kernel of each contribution to the discussion by any of the participants in chronological order. These narrative summaries (total length 11’700 words) where then analysed thematically.^30^ This is a very widespread approach to analyse textual data, which consists in identifying themes in the data based on a conceptual framework and a codebook, which can be developed inductively or deductively. In our case, we adopted an inductive approach, whereby our core team organised meetings and discussed the content of the data collected. Starting from the data themselves, a list of themes and subthemes capable of condensing the main topics therein was developed. Based on these themes and subthemes, we then wrote a narrative summary of the main issues that we derived from the data. The results of such analysis were then shared with the participants between April and May 2022 to gain their feedback, observations or comments. Participants were also invited to collaborate in the writing of this article, and they are thus amongst the co-authors or listed in the acknowledgements. Figure 1 summarises the structure we gave to our modified Delphi process, highlighting the different rounds of interactions with the involved experts.

## Results

We present the main findings of our study in a narrative form, based on a descriptive analysis of the responses to the preliminary questionnaire (reported in tables at the beginning of each section), the thematic analysis of the qualitative data recorded during the round tables and the additional feedback provided afterwards by the Delphi panellists. Direct quotes from the recordings made during the workshop are not included, since those represented only an intermediate product of our modified Delphi process, before the final round of feedback provided by panellists.

### Techniques to facilitate data sharing practices

When asked in the preliminary questionnaire (see 1.1 in Table 1), most participants agreed that some progress in promoting health data sharing has already been happening in Switzerland. When prompted about this issue at the workshop, there was agreement that (1) progress has been achieved mostly in terms of ‘putting the topic on the agenda of several institutions’, whereas much less progress has been made in practice (e.g., in terms of cutting-edge projects developed thanks to data-sharing); (2) progress of data sharing differs substantially depending on the contexts. For example, participants commented that SPHN has done a lot to promote data sharing of routinely collected clinical data, and that also with respect to cancer registry data there were concrete steps ahead thanks to the recent legislative reform and the discussion on data access it sparked. However, other sectors of the health data landscape lag behind. Panellists also agreed on the statement that cultural and technological changes like promoting data sharing take a lot of time: it is necessary to upgrade data infrastructures, find consensus amongst stakeholders, and clarify (and then meet) all the necessary legal and security requirements. Given the efforts that implementing these elements entails, all panellists underlined that progress in data sharing can only be measured by having a long-term perspective. In fact, the sectors that are starting to show promising inclinations towards data sharing are those that have benefitted from financial investments and interdisciplinary support (e.g., SPHN in respect to secondary use of health for biomedical research), political support (e.g., Cancer registries, which were given a legal basis to collect data for public health surveillance all over the country) or institutional commitment (e.g., some health insurances have started sharing their data to help conduct healthcare service research).

**Table 1.** Questionnaire results regarding the opinions related to the topic “Techniques to facilitate data sharing practices”.

	Questionnaire items	Answers in %				
		Strongly disagree	Somewhat disagree	Neutral	Somewhat agree	Strongly agree
1.1	In your opinion, there has been progress with respect to health data sharing in Switzerland in the last few years	8	23	0	46	23
1.2	In order to promote data sharing…					
	a)… data-collectors should receive authorship or other collaboration opportunities in return for the datasets they share	29	29	0	29	21
	b)…the academic reward mechanism for those who share data should be improved	0	8	23	38	31
	c)… there should be financial compensations for data-collectors for data sharing activities	0	23	31	31	15
		Law is much more problematic	Law is more problematic	Equally problematic	Application is more problematic	Application is much more problematic
1.3	With respect to data sharing, is the lack-of-clarity in data protection law itself more problematic or the lack-of-clarity in the application of the law?	0	8	31	46	15
		Strongly disagree	Somewhat disagree	Neutral	Somewhat agree	Strongly agree
1.4	It is reasonable to request equivalent data in exchange for one's own dataset, in particular when collaborating with external researchers	23	23	31	15	8
1.5	I believe I will be more inclined to share my data if there are standardised legally-binding data sharing agreement templates that stipulate clear data ownership, processing and publication rules to protect my interests and those of data subjects	15	0	8	23	54
1.6	I believe I will be more inclined to share my data if I can keep an oversight on the data processing activities of data recipients to prevent any misinterpretation or misuse	8	23	8	38	23
1.7	I believe that better…………with other researchers/stakeholders would incentivise data-collectors to share ‘their’ data					
	a)… communication…	0	0	15	85	0
	b)…coordination…	0	0	16	69	15
	c)…reciprocal knowledge…	0	0	16	69	15
1.8	I agree that data collectors should keep control over ‘their’ data.	0	16	15	46	23
*1.9*	*If I want to answer a research question using already existing health data sources (e.g., hospital databases or registers), it is difficult to find these data sources*	0	33	25	25	17
1.10	If I want to answer a research question using already existing health data sources (e.g., hospital databases or registers), it is difficult to gain access to these data sources	0	8	0	67	25
1.11	Limiting the discretion of data-collectors (i.e., the liberty of data-collectors to decide ‘freely’ who can get access to ‘their’ data and who cannot) would facilitate having access to existing data sources	8	8	23	53	8
1.12	I believe it would be easier to use existing data sources, if they were all collected with the prospect of being re-used at a later stage by external researchers or institutions	8	0	0	15	77
		Definitely more formal collaborations	Rather more formal collaborations	Neutral	Rather more informal contacts	Definitely more informal contacts
1.13	To favour data sharing between different stakeholders and to build trust, we would need more formal collaborations (e.g., information about what other researchers/stakeholders are doing) or more informal contacts (e.g., meeting, networking events etc.)	15	8	46	23	8

*Note*. The total number of respondents were 13. In 1.9 and 1.10, one person did not respond. Percentages are rounded up.

Sharing health data more efficiently in Switzerland necessitates knowledge of existing data sources and the accessibility requirements may vary greatly for every data source. A few panellists explained that finding data sources can be easy or difficult, depending on the sector (as highlighted above) (see also 1.9) and on experience as well as existing personal network of the single researchers trying to find the data. This is also connected to the very fragmented and decentralised nature of Swiss healthcare. They also highlighted that – even once found – it remains difficult to actually access that data and therefore, it is important to offer two kinds of incentives: (1) to promote ‘willingness-to-share’ and make data more findable in the first place; (2) to reward the actual act of sharing data. As to the latter issue (i.e., what incentives to give so that data are actually shared), the idea was advanced that it should not be a taboo to financially remunerate those who share the data as a compensation for the efforts to make data reusable. This could also help to make the conditions for data access more transparent (i.e., as long as one pays, then data can be provided and reused), but it would depend on how this is implemented – e.g., certain data providers might still deny sharing data based on the research question/analysis that those who request access want to carry out, or for the reason of not automatically being a co-author on the publication of the resulting study. As to the incentives to promote ‘willingness-to-share’, four ideas were shared by panellists. First, promote the creation of a political discourse that those who collect health data from the public (e.g., social health insurance) should share such data for research, when this promotes the ‘common good’. Second, incentivise hospitals to share those datasets that are of interest for research by ensuring that they receive better publicity and more public visibility. Third, address the ‘data protectionism’ culture of academia by providing career incentives to researchers who share their datasets. Fourth, elaborate a more precise legal framework to guide and reassure data providers in the sharing of data. This could be done, for example, by including data sharing activities as an additional criterion in the academic reward system. Another element discussed to promote a ‘data sharing culture’ was to build trust amongst stakeholders. Whether it happens through formal or informal collaborations (related to 1.13 in Table 1) was deemed relatively unimportant. Participants underlined that the core effort is to invest on the ‘relational’ (rather than only ‘technical’) aspects of the interaction between stakeholders that could share data, for example by promoting open communication between stakeholders and by increasing trust through the advertisement of ‘good/successful examples’ of collaborations facilitated by data sharing (see also 1.7a,b,c). During the discussion about incentives to favour data sharing, there was opposition to the idea of giving authorship in return for the act of sharing data *per se*. Scientific collaborations should be promoted, and co-authorship should not be ostracised, but it cannot be obtained by simply providing data, as discussed by recent Swiss guidelines.^31^ In the research context in particular, rewarding those who make a project possible is an important incentive, which can be reached – depending on the circumstances – by monetary compensation of the institution/researcher providing data, by mentioning of the data source or – in case of substantial contribution –with co-authorship.

Data controllers (i.e., those who actually manage the datasets)^3^ – in particular when these are institutional actors (e.g., hospitals or health insurers) instead of researchers – may often resist data sharing due to (potentially legitimate) concerns. Many panellists had indicated in the preliminary questionnaire that they agreed that data controllers have to retain control and oversight over ‘their’ data (see 1.6 and 1.8), also because data controllers have obligations towards data subjects (i.e., the people where the data come from) to ensure data is handled properly. However, when discussing these issues in the workshop, the participants specified what kind of ‘oversight’ and ‘control’ are necessary to ensure data sharing. The panellists highlighted that the core element to overcome concerns about data sharing – especially in when hospitals are sharing data for further research uses – is to ensure that everything is done according to the legal, ethical and data security requirements when data are transferred (see also 1.5), so that there is more certainty that sharing data entails minimal risks.

### Moving ahead in the ethico-legal domain

With respect to the ethico-legal domain, the role and implementation of patient involvement was heavily discussed. Specifically, it was debated how current practices for data uses in Switzerland often rely on individual consent by data subjects, when their data are (re)used (e.g., for research) – see also 2.1–2.7 in Table 2. This topic is particularly important given the specific rules that the Human Research Act^32^ sets for the secondary use of data for research purposes in the Swiss context.^17^ Indeed, Switzerland differs from the European Union in that the latter sets many legal rules on the secondary use of data for research in their overarching regulation on data protection (the GDPR), whereas the former has more detailed rules on this issue in the sector-specific legislation on human research, i.e., the Human Research Act.

**Table 2.** Questionnaire results regarding the opinions related to the topic “moving ahead in the ethico-legal domain”.

	Questionnaire items	Answers in %				
		Strongly disagree	Somewhat disagree	Neutral	Somewhat agree	Strongly agree
2.1	Every time health data from patients are reused for research, patients should be individually informed about that specific analysis	46	15	8	8	23
2.2	Retrospective research (i.e., using already existing data) should only go either through research ethics approval or data protection approval	18	8	8	33	33
2.3	Total anonymisation’ of patient health data should be the main ethical requirement for conducting analysis of such data	31	23	15	31	0
2.4	It is clear what requirements researchers and/or data-analysers need to fulfil to ‘anonymise’ patient data in Switzerland	31	46	8	15	0
		Never	Seldom	Sometimes	Often	Almost always
2.5	The legal requirements about consent and/or data-protection end up generating legalistic and contractual “battles” rather than ensuring ethical and privacy-protecting research	0	15	31	46	8
		Strongly disagree	Somewhat disagree	Neutral	Somewhat agree	Strongly agree
2.6	In Switzerland, we should explore alternative ways (e.g., public informational campaigns about the major objectives why health data are analysed) to keep data-subjects informed and aware of what their health data are used for or what are the potential benefits and risks	0	0	15	23	62
2.7	There is a need to change the consent model for research projects using already existing datasets from the opt-in to the opt-out model	0	23	0	46	31

*Note*. The total number of respondents were 13. In 2.2 and 2.5, one person did not respond. Percentages are rounded up.

Some panellists were very sceptical about the current practice of asking individuals if they agree to give general consent before data are (re)used, and they doubt whether such consent would truly satisfy a core feature of informed consent (i.e., data subjects are genuinely involved in knowing what is done with health data). A few other panellists thought that providing information at the individual level and collecting consent have limits that could be overcome if these activities are properly implemented and financed. Notably, some remarked that proper implementation would lead to the problem that financial support, time and a build-up of the data infrastructure (e.g., to monitor who has consented to the re-use of her data) would be needed. Moreover, it was observed that data protection law currently places a lot of emphasis on consent (unless data are anonymised), thus making it difficult to deviate from this focus in practices.

Overall, there was agreement on two issues. First, data-subjects should be kept involved in the use of ‘their’ data (if they want to), and that new ways to do this can be explored. For example, through informative campaign about data usages, or through better communicating to the public what kind of research is done and explaining its expected utility and results. Indeed, fostering direct communication and trust with the general public would be key in shifting from the over-protective narrative often heard in Switzerland (referring to hypothetical and very marginal privacy risks, etc.) towards a narrative of promoting re-use of data for the common good. However, such communication with the public and patient organisations needs to be conducted by trusted institutions. Secondly, individual informed consent for data usage, if kept as the main ethico-legal condition to (re)use data cannot turn into an ‘alibi consent’, i.e., an instrument of mere legal compliance that data controllers develop only in order to be ‘okay’ with respect to data protection law, but where the idea of ‘genuinely informing’ the data-subject gets overshadowed. A potential solution to this could be the development of a more innovative informed consent model (i.e., where people are still asked to provide consent, but for broad category of research, rather than for single projects).

Another central component of the debate were data protection aspects of health information exchange. Amongst participants, there was a widespread perception that institutions examine the data protection implication of data exchanges according to different standards. This is considered both a cause and a consequence of the Swiss managerial and societal ‘risk-focus’ with respect to data. Panellists perceived that – with a view to a more and more systematic secondary use of data – there are a lot of fears related to data misuses and to data breaches, which result in stakeholders exchanging data being very restrictive and ‘picky’ in how data agreements (e.g., data transfer agreements) are designed. To overcome this, panellists underlined once again the importance of communicating between stakeholders to reach common standards, so that each negotiation to exchange data is not too much dependent on the ‘goodwill’ of single parties involved. Such dialogue would have to include also legal experts, since data protection remains a very complicated field where legal expertise is needed. Some panellist mentioned SPHN as a positive example – with respect to clinical data from hospitals – how this can be achieved. By fostering dialogue between parties, SPHN has created standard templates for legal agreements regarding data exchanges in multi-centric research projects.^4^ There was no agreement in how a similar evolution could be achieved with respect to data held by social health insurance. Some suggested promoting the narrative that such datasets (being social health insurance obligatory for all residents) are a form of ‘public good’. However, others highlighted that this narrative would be difficult to introduce, because e.g., it is unsure which institutions could do it.

Lastly, panellists discussed the problematic topic of health data anonymisation. First, it was highlighted that data protection law and HRA^32^ define anonymisation in a way that is very difficult to operationalise (see also 2.4), especially because of the blurry distinction between anonymised data (i.e., where it is impossible – or possible but only with disproportionate efforts – to re-identify the original data-subject) and pseudonymised/coded data (where the identifying characteristics of the data are replaced by a code, in such a way as to make to make the data unidentifiable without possession of the code). Panellists argued that the law should focus on the crucial difference between identifying datasets (e.g., data where the identity of the data subject is evident) and pseudonymised/coded ones, since with the former the privacy of data subjects is evidently exposed, whereas with the latter is protected, albeit not anonymised. It was underlined that – even if it was possible to totally anonymise data – this would not ‘per se’ make a project ethical (see also 2.3). Also mentioned was the fact that anonymisation hampers a number of research purposes, since it may make datasets quite useless in most cases, whereas pseudonymised/coded datasets would still allow linkage. All panellists agreed that lawyers and data processors (e.g., researchers) should agree on a renewed conception of ‘sufficient anonymisation’ for certain uses of data, e.g., research. This should allow to add data over time or even start projects where data is linked to other ‘sufficiently anonymised’ datasets, without fear of infringing data protection provisions.

### Policy changes for improving data governance

The third topic addressed in our study concerned more general policy changes that are desirable to improve health data governance. In this regard, a commonly shared point of departure for the discussion amongst panellists was that Switzerland is still lagging behind in terms of coordination and reciprocal knowledge of the different stakeholders composing the health data landscape (see also 3.2a,b,c in Table 3). To overcome this, it was argued that more proactive platforms for dialogues should be promoted. Different stakeholders, namely researchers intending to access health data, institutions like hospitals or insurance companies managing datasets and policymakers requiring data to inform public policy, may develop a collaborative approach before the actual need to exchange data for a specific project emerges. Currently, this happens only to a certain extent and in some contexts (e.g., with the work of SPHN, the preconditions to facilitate the exchange of data for research purposes from different hospitals has been improving), whereas in others the ‘risk-focus’ still dominates. This entails a tendency to ‘put the data protection law(yers) forward’ and adopt protective attitudes at the forefront of negotiations, thus often creating complications. Panellists acknowledged that overcoming this ‘defensive attitude’ may be difficult in some cases. For instance, hospitals have many justified fears about sharing data, due to their responsibility for handling the data and the risk of bad publicity in case of data breaches. Nevertheless, panellists highlighted the importance of creating proactive platforms for dialogues, for example, by referring to an initiative of the Swiss Academy of Medical Sciences (SAMS), which received the mandate to set up a National Coordination Platform for Clinical Research to help different stakeholders in this field to align priorities on how to conduct research and exchange clinical health data.^33^

**Table 3.** Questionnaire results regarding the opinions related to the topic “policy changes for improving data governance”.

	Questionnaire items	Answers in%				
		Definitely national priorities	Rather national priorities	Neutral	Rather individual initiative	Definitely individual initiative
3.1	It is better to have clarity and indications of what the national priorities are concerning what health data should be collected (or be made more easily re-usable), or it is better to leave it to the initiative of single researchers and/or institutions	38	16	38	8	0
		Strongly disagree	Somewhat disagree	Neutral	Somewhat agree	Strongly agree
3.2	There is enough information ‘going around’ concerning…					
	a) …the data sources in Switzerland are	8	54	15	23	0
	b) …the quality of data sources in Switzerland	8	69	15	8	0
	c) …the potential uses for data sources in Switzerland	8	69	15	8	0
3.3	I believe that data protection authorities should be the only institutions required to approve non-interventional research projects using already existing datasets	31	23	7	31	8
3.4	Having a unique personal identifier that is used in all datasets in Switzerland would be ideal.	15	0	0	23	62
3.5	More political commitment and public information should be dedicated to discussions about the unique personal identifier	8	0	15	31	46
		Definitely institutionalised ways	Rather institutionalised ways	Neutral	Rather political/negotiation powers	Definitely political/negotiation powers
3.6	In Switzerland, institutionalised and/or standardised ways should be developed to favour data access OR data access should remain a matter of political and/or negotiation powers	59	33	8	0	0
		Strongly disagree	Somewhat disagree	Neutral	Somewhat agree	Strongly agree
3.7	It would be a sensible investment to develop a ‘National Data Center’ that manages and provides data to researchers	31	15	8	31	15

*Note*. The total number of respondents were 13. In 3.6 and 2.5, one person did not respond. Percentages are rounded up.

Throughout the roundtables – but especially on the last one dedicated to future policy changes – it was remarked several times that it is important for policymakers to appreciate that a certain degree of differentiation is necessary: specific solutions have to be developed for the different data-environments (e.g., hospital databases, health insurance databases, research-lead databases, and public health or statistical databases). Such differentiations would also help to identify any data-environments that should be prioritised. In this respect, it was discussed whether it would be appropriate for Switzerland to define specific national priorities for the development of the data landscape (see 3.1). Panellists were divided about this, but there was agreement about two points. First, they agreed that the reuse of already existing data somehow collected through public funding (e.g., those managed by obligatory social health insurance or public hospitals) should be promoted, also to foster the idea that there are certain data to be conceived as public good. Second, it was remarked that reflecting on national priorities (whether they are thereafter established or not) would help to shed light on those data-environments which need improving, especially in terms of creating a data infrastructure that increases transparency. As an example, participants mentioned the general practitioner and ambulatory contexts, about which nowadays too little is known/researched.

Two further pressing policy issues were debated. First, whether it would be appropriate to put the introduction of a unique patient identifier that would help to link data across databases high on the political agenda. In respect to research, panellists generally agreed that this would be desirable (see 3.4 and 3.5 Table 3), but they questioned if this is politically viable. Second, it was debated if some kind of ‘data centre’ with a specific competence for the health sector should be created (see also 3.7 Table 3). In this respect, during the discussion everyone rejected the idea of a centralised data storage or warehouse. However, many panellists agreed that it would be desirable to have some kind of ‘accredited gatekeeper’ or ‘data broker’ that could coordinate interactions and data exchanges between different stakeholders or that could maybe be used as a trusted centre to perform record linkages (or merge data from different sources) without anyone external having access to the keys used to link data. But even in this respect, panellists underscored that establishing such ‘data centre’ could meet the resistance of some stakeholders.

## Discussion

In this study, we collected views and assessments from a set of stakeholders to appraise the state of the Swiss health data landscape and suggest concrete steps for its future developments. Three comprehensive topics were addressed, including that of data-sharing, ethico-legal challenges and policy changes. The findings from our study provide several inputs at a critical moment of the development of the Swiss health data infrastructure and governance, especially concerning the sharing and reuse of data for research purposes.

The first crucial input concerns the necessity to understand that the development of the health data landscape of a country is necessarily a long-term process. Any advancement in this field is gradual, also because it requires to change not only the technical infrastructure, but also the mentality of stakeholders involved and of society at large. For example, a report from the Swiss Health Observatory on the evolution of eHealth solutions in the outpatient sector in Switzerland shows clearly that improvement takes place in a gradual rather than exponential way.^34^ In the past few years, many projects were started with a strong *promissory* emphasis. For example, the reform of Swiss cancer registries was portrayed as an historic change, since it created for the first time an obligation to systematically record cancer related data all over the country. Or else, the creation of the infrastructure for the use of an interoperable EPD to collect patient data has been accompanied the expectation that it would (from the moment it is activated) bring Switzerland in the digital age. However, neither of these initiatives has solved all the issues in their respective parts of the national data landscape. The reform of cancer registries was met with very sceptical reactions by some stakeholders,^35,36^ and a recent study has highlighted the reform created hurdles to collect certain types of data, which would be very useful for research and public surveillance purposes.^37^ Similarly, it remains difficult to determine to what extent the EPD reached success in facilitating the reuse of data in clinical care.^38,39^ Whereas it is true that projects like these determine advancements in the sharing of health data in their respective context (clinical care for EPD and public health surveillance for the cancer registry) and also for data sharing more broadly (since the potential reuse of data from these sources is being discussed), it cannot be expected that they will solve all problems or will be implemented without barriers. The development of the various components of the health data infrastructure and of data sharing in the research context depends on a long-term commitment and, even after an infrastructure or governance-building project becomes operative, much work remains to be done to optimise implementation.

Adopting a long term perspective is also connected to the need of changes of a cultural nature to improve the sharing of data in the health data landscape. Indeed, alongside the build-up of a technical infrastructure, it is also necessary to consider the readiness of those operating the infrastructure (e.g., researchers, database managers, or healthcare workers), whose advancement might proceed at a slower pace. Previous literature on data sharing has already highlighted how socio-cultural hurdles are difficult to overcome (e.g.,^40^). Two relevant cultural changes were mentioned in our study. First, it was highlighted that Switzerland needs not only to create incentives that are given when data are actually shared between institutions, but also – and more importantly – a series of incentives that stimulate the *predisposition* towards sharing. For example, it was remarked that one such incentive for hospitals could be the guarantee that those clinics which ‘open up’ their datasets more easily are given special public recognition or that it is ensured that the insights discovered through analysis of their data are fed-back for improving services. This would have to be combined with a clear definition of data governance responsibility within institutions, and by the promotion of a cultural chance in society at large, whereby the value of sharing data for research is appreciated.

Second, our study remarked that Switzerland needs to escape from a detrimental ‘risk-focus’ concerning data exchanges, whereby many institutions are fearful of (even marginal) risks that could derive from increased data flows. This ‘risk-focus’ was described as specific of Switzerland, but the fear that even data processing entailing minimal levels of risk is hampered by over-restrictive standards is shared internationally.^41^ It is undeniable that – with the increased datafication of healthcare and research – there are risks entailed by the exchange of data, and many safeguards (both technical and ethico-legal – such as having solid legal basis for processing data) are necessary. However, it would be important to discover whether such ‘risk-focus' derives from these worries or if it is rather connected to the fear of institutions (which host data) or researchers (who created datasets or cohorts) that opening their datasets could expose *them* (rather than the data-subjects) to ‘reputation’ risks – e.g., by revealing poor data management or – for hospital – showing patterns of under/over-treatment. Even when these risks are considered, they are certainly of a different nature compared to privacy risks for data subjects. For the future, it would thus be important to survey with data controllers, where exactly their protective attitude towards sharing data derives from and how their (excessive) risk-adverse behaviour can be changed. It would be important to compare the sources of fear of data controllers and data recipients (i.e., those with whom data are shared) with those of data subjects. The latter have been investigated by two recently published surveys with the Swiss population.^42,43^ If fears of data subjects and those of data controllers are significantly misaligned, there might be the risk that the restrictiveness of data controllers is depicted as a way to defend the privacy of data subjects, but in reality it is a way to defend their own interests.

Another important input of our study concerns legal and ethical aspects of data governance. It is by no means a particularity of the Swiss context that the datafication of healthcare and research sector is generating challenges in this sense. Our results confirm what in the literature has been described as the deterioration of the consent or anonymise approach.^44^ Before the onset of the digital age, it was considered ethically and legally acceptable that health data processing, especially for research purposes, is allowed if consent is obtained or if the data are anonymised. This approach started being challenged as it became evident that full anonymisation is a chimera, and that consent is often impracticable and may be a disproportionate burden, when the same dataset is re-used for multiple times for different projects. To move ahead, there is still the need to keep data-subjects informed about what happens with their health data. This can be achieved either by reinforcing and modernising the infrastructure for collecting consent, exploring the idea of presumed consent, or by reaching citizens in alternative manners (e.g., informational campaigns about the research projects conducted through the secondary use of data). As an alternative, there have been attempts in Switzerland to implement the concept of general consent, whereby individuals are still asked for their permission to process their data, but for not-yet-fully-defined research projects rather than single studies.^45,46^ A recent report by the Federal Office of Public Health on the secondary use of health data also insisted that the element of consent should remain central, but that the system for the collection of consent should be modernised.^47^ Proposed solutions included the idea of an electronic consent and the possibility of allowing citizens to donate their data for secondary uses in the public interests. Although the Swiss approach to health data governance remains heavily invested in the idea of individual control over data, there are ways to process health data without consent. This can happen if a specific law authorises it, or – in the context of research – if a series of requirements are satisfied, in line with Art. 34 of the HRA.^32^ From an ethical perspective, a possibility is that of exploring the implementation of some form of *community consent*, whereby those who use health data (e.g., researchers, sponsors or health authorities) “engage with participants and communities throughout the life-cycle of the research”.^48^ This kind of activities may not constitute a legal basis for the processing of health data in the current Swiss legal context, but they would be important to increase the social legitimacy of data processing activities and to increase the trust of the population towards the use of health data.

Our results indicate also that data anonymisation is a challenge and that data protection aspects are often tackled in a protective and watchful manner by the parties involved in processing and exchanging data. This leads to one key consideration, namely that it is important to promote proactive interaction between data protection experts and institutions who process data. By proactive, we refer to the need that the interaction should take place before data are exchanged and should be aimed at preventing problems, rather than at negotiating solutions once problems have emerged (e.g., because data are to be exchanged). Two important issues should be at the core of this interaction. First, as we suggested in another study,^18^ it would be necessary to agree on a definition of *sufficient anonymisation*, or – to put it differently – ‘what levels of data security (e.g., by using pseudonyms or the elimination of direct identifiers, such as date of birth or residence) are necessary to proceed with large data analysis (e.g., research studies in the field of personalised health) in a legally compliant way?’. One potential answer to this question has recently (May 2022) been elaborated by the Data De-identification Project Task Force of the SPHN.^49^ Second, it would be relevant to agree on best practices to follow when data from different databases are linked, so that the single data controllers do not have to fear privacy violation, which would make them reluctant to share their data. A starting point for the latter issue could be a recently published report on the topic,^50^ the experience that the Federal Statistical Office is gaining in providing data-linkage services for third parties,^51^ or the experience with probabilistic linkage methods being accumulated by some research centres in Switzerland.^52^ A positive development in this sense is also a recently promoted political motion^53^ which is aimed at creating the necessary legal basis to have a unique patient identifier used by all actors collecting healthcare data, which may later facilitate linkage. Indeed, the Federal Council has now officially recognised that improving the conditions for data linkage is a crucial component of the future system for the secondary use of health data in Switzerland.^47^

### Limitations

Our study has limitations. For a start, its findings are not generalisable to mirror the views of all stakeholders in the Swiss health data landscape, since the number of participants was limited and some players (e.g., social health insurances) were not represented. This is a generally acknowledged limitation of any Delphi process, which is sometimes referred to as reliability in the methodological literature (i.e., would have a different panel reached different conclusions?).^54^ It was also in line with the objectives of this study, which is of an exploratory, rather than explanatory nature. Further research can be elaborated to confront our findings with those generated by different types of participating stakeholders, e.g., hospital managers and health insurance companies. Another limitation is that we adapted the Delphi methodology, from its classical form. At the same time, we took care of preserving the following essential features of this methodology. First, there was the presence of a step in the process where involved parties could provide their input anonymously (for us, the initial questionnaire). Second, we maintained the possibility for the experts participating to iteratively provide input during the process (in our case, input was provided with the initial replies to the questionnaire, then again during the round-tables, and then afterwards when findings were fed-back to participants, who also contributed to writing this article). Third, we maintained the element of having a structured interaction, which our core team took care of organising and maintaining during the whole process. A related limitation concerns the issue of establishing how to define “agreement”. One of the fundamental feature of a Delphi methodology consists findings areas of consensus between experts, thus begging for a definition of the minimal level of agreement necessary to determine that there is agreement on an issue. This problem emerges especially in respect to traditional Delphi-processes which are structured only through reiterated questionnaires (thus requiring a quantitative definition of agreement). In our case, we refrained from a precise definition, but we relied on: (1) repetitively inviting Delphi panellists during the round tables – in cases where it seemed that there was general consensus on an issue – to raise objections; (2) submitting our analysis of the questionnaire and the discussion in the roundtables to panellists for feedback, so that they could raise objections to any point on which we deemed consensus was reached or considered to be ‘uncontroversial’ in our analysis. Moreover, our application of the methodology (see the section “Study design and methodology”) as a modified Delphi is less concerned with agreement and more with exposing “the differing positions advocated and the principal pro and con arguments for those positions”.^55^ The final limitation of our study is that, although we analysed some concrete solutions and some policy proposals to address them, we did not explore which stakeholders would most benefit from their implementations. This can be addressed in further studies that test the resonance of our results with quantitative designs and representative samples.

## Conclusions

In this article, we reflected on the current state and especially the future development of the Swiss health data landscape based on a multi-stage modified Delphi process. This topic is bound to remain a central concern in health policy in Switzerland, given that healthcare has been listed as core focus-theme in the Digital Switzerland Strategy 2023 and that the creation of a special law for the secondary use of data is under discussion.^56^ However, given that the datafication of healthcare and biomedical research is a phenomenon that concerns the whole world and that promoting digitalisation remains a policy priority not only for Switzerland, our study provides inputs that can be relevant also for other contexts. For example, the European Union has also been grappling with the problems related to the secondary use of health and is thus equally looking for solutions.^57^ There are two aspects in particular that we want to stress, and that we invite other countries to consider as well. First, the fact that the progress of the health data landscape is dependent on a great deal of ‘non-technical’ aspects: from the necessity of a culture of openness towards data sharing and the necessity of developing best practices and standard procedures on how to *operate* in an ethically, legally and societally acceptable way the technological infrastructure which is developed. Second, the importance of starting a proactive debate between data-holders and those who want to access and reuse the data (e.g., researchers), rather than limiting interactions to the actual moment when data are needed. Such a debate would need the involvement of data protection experts, with the objective of developing ways how privacy law can be efficaciously *operationalised* with respect to health data exchanges.

## Notes

1. For more details, see: https://sphn.ch/network/data-coordination-center/

2. Several experts have experience in more than one field.

3. We use the term ‘data controllers’, since this is a widespread legal term to indicate those who control a certain dataset and decide upon the purposes and the means how data are processed. Cfr. Art. 5 lit. j of the revised Federal Law on Data Protection in Switzerland, or art. 4 para. 7 of the General Data Protection Regulation (GDPR) in the European Union.

4. Available at: https://sphn.ch/services/dtua/
